# Supplementary material for: RAAC panels can suddenly collapse before any warning of corrosion-induced surface cracking
Source: Npj Mater Degrad. 2025 May 7;9(1):44. doi: 10.1038/s41529-025-00596-5 (PMC12058510; doi:10.1038/s41529-025-00596-5)
Supplement: Supplementary file 1 — Supplementary Information [file 41529_2025_596_MOESM1_ESM.pdf]

Supplementary Information

Evžen Korec[1,2], Peter Grassl[3,4], Milan Jirásek[5], Hong S. Wong[1], Emilio Martínez-Pañeda[2,*]

[1]Department of Civil and Environmental Engineering, Imperial College London, Exhibition Road, London, SW7 2AZ, United Kingdom.
[2]Department of Engineering Science, University of Oxford, Parks Road, Oxford, OX1 3PJ, United Kingdom.
[3]James Watt School of Engineering, Rankine Building, Glasgow, G12 8LT, United Kingdom.
[4]Glasgow Computational Engineering Centre (GCEC), University of Glasgow, Glasgow, G12 8LT, United Kingdom.
[5]Department of Mechanics, Faculty of Civil Engineering, Czech Technical University in Prague, Thákurova 7, Prague - 6, 166 29, Czech Republic.

1 Notes on the numerical solution

The model was numerically solved in Matlab by iterating the value of u_c so that both equations (7), (12) and (13) are fulfilled and thus pressure residual $R = p_1 - p_2 = 0$. To this end, fzero function [1] using derivative-free methods of bisection, secant, and inverse quadratic interpolation methods is employed. The physically admissible solution is $u_c \geq 0$. However, in the initial stage of corrosion, most of the rust accumulates in pore space rather than in the dense rust layer which does not exert any pressure yet as $t_{cor} \leq \kappa t_r$. Though physically incorrect, the mathematical solution of the model is in this case $u_c \leq 0$. To correct this discrepancy and speed up the calculation, we employ a convenient property of residual R, which was found to be a monotonically decreasing function in the performed case studies. Thus, condition $R \leq 0$ for $u_c = 0$ is equivalent with $u_c \leq 0$ for $R = 0$. In the provided code, the physically correct solution $u_c = 0$ is assigned if $R \leq 0$ is tested for $u_c = 0$. To solve ordinary differential equation (13), bvp4c code based on a finite difference method with Lobatto IIIa collocation formula [2] was used[1].

[1]All developed codes are freely available at https://mechmat.web.ox.ac.uk/.

2 Notes on the focus of the model and some simplifying assumptions

The aim of this manuscript is to investigate the period of time during which rebar corrosion in RAAC panels remains concealed. This means that the focus is on the propagation phase of corrosion, i.e. after the initiation of corrosion at the steel surface. Therefore, we do not investigate the processes that lead to the initiation of corrosion, in particular the transport of moisture and aggressive corrosion-initiating species such as chlorides and carbon dioxide from the surface of the RAAC panels to steel rebars. Due to their high porosity, RAAC panels are highly permeable to water and aggressive species [3]. As a result, the time to corrosion initiation can be significantly shorter than for standard-density concrete, where it typically dominates over the corrosion propagation period. The mathematical description of moisture, carbon dioxide and chloride transport can be easily added to the proposed model, as we did in our previous studies [4, 5], and the possible non-uniformity of corrosion expected in a chloride-rich environment can be taken into account, as in [5]. The high permeability of RAAC also causes that the concrete cover zone of panels can be expected to be highly carbonated [3]. This means that the corrosion process is likely to be relatively uniform along the rebar surface. We therefore make the simplifying assumption of uniform corrosion in the proposed model. To reduce the complexity of the model and the associated uncertainties, the complex electrochemical corrosion reactions are not explicitly simulated and the constant value of the corrosion current density $i_a = 1$ µA/cm^2 is assumed. This is admittedly a simplification and the value of the current density is strongly influenced by the water saturation of the concrete [6] and the availability of oxygen [7]. However, the value of the current density of about 1 µA/cm^2 has been found to be typical for standard-density concrete corroding under variable conditions [7–11]. As there are currently no experimental or computational results available on the magnitude of the corrosion current in RAAC panels, the value chosen is considered to be a reasonable approximation.

3 Choice of model parameters

The parameter values of rust and concrete related to the transport of iron ions are summarised in Supplementary Table 1. It should be noted that to this date, no experimental measurements of these constants for RAAC have been reported in the literature. Thus, the presented values are based on the measurements or estimates reported for standard concrete. Although Ansari et al. [12] considered the diffusivity of iron ions in rust D_r in the order of magnitude of 10^{-11} to 10^{-10} m^{2}s^{-1}, D_r is currently not well experimentally documented. For this reason, we conservatively considered the upper bound of $D_r = D_w$, i.e. we set the diffusivity of iron ions in rust to the same value as in water. It is most likely that $D_r < D_w$ and the assumption that $D_r = D_w$ provides conservative estimates (upper bounds) of the critical corrosion penetration t_{crit} (i.e. of the corroded steel thickness leading to the first surface cracks). The remaining parameters were considered in the same way as in our previous studies on standard concrete [5, 13, 14]. The values of Young's modulus and

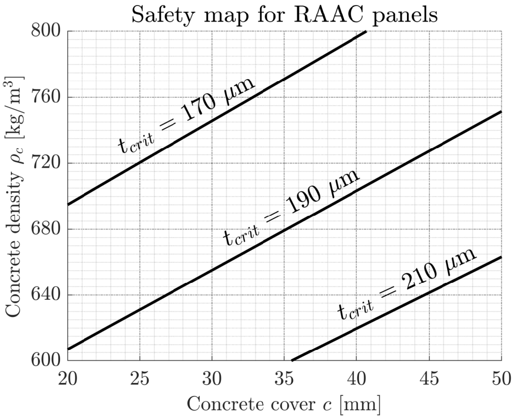

Supplementary Figure 1: **The impact of chemical parameters of the model on the critical corrosion penetration.** Values of calculated t_{crit} are affected by the choice of chemical parameters of the model, especially the reaction rate $k_r^{II\to III}$, which are currently unknown for RAAC. A significant increase in predicted t_{crit} is observed (see Fig. 2c in the main manuscript) if the lower value of $k_r^{II\to III} = 0.01$ mol^{-1}m^{3}s^{-1} resulting in a slower rust precipitation is considered.

Poisson's ratio of rust are infamously scattered in the current literature. Thus, we considered two intermediate values within the range reported by Zhao and Jin [15], which were found to lead to reasonable estimates of corrosion-induced crack width in [5, 13, 14]. It was proposed that the molar volume ratio of rust and steel κ depends on the magnitude of corrosion current density [13]. Since the main objective of this study is natural corrosion in RAAC panels during which i_a is supposedly smaller than 1 µA/cm^2, as typically reported for standard concrete [7–11], we considered the value of κ corresponding to $i_a = 1$ µA/cm^2 according to our previous work [13]. The implied value of $\kappa = 3.17$ is in the range of the values reported in the literature [15–17] for commonly present iron oxides and iron hydroxy-oxides in rust. It should be noted that our previous work [13] was based on the analysis of impressed current tests with chloride-induced corrosion. The presence of chlorides is known to affect the nature of the emerging corrosion products [18]. Chloride-induced corrosion is known to produce the rust with the lowest density and therefore the highest κ, such as akaganeite ($\beta -$ FeOOH). As discussed earlier, corrosion in RAAC panels is likely to be carbonation-induced. Therefore, consideration of κ, which is characteristic of chloride-induced corrosion, may lead to an underestimation of the predicted critical corrosion penetration. Further research is required to characterise the types of rust formed during RAAC corrosion. The estimate of the reaction rate $k_r^{II\to III}$ governing the rate of oxidation of Fe^{2+} to Fe^{3+} ions and thus implicitly the rate of rust precipitation, was adopted from the modelling study on carbonated standard concrete [19].

It should be noted the predicted t_{crit} is strongly affected by the choice of $k_r^{II\to III}$. As can be observed in Supplementary Figure 1, predicted t_{crit} increases significantly if lower value of $k_r^{II\to III} = 0.01$ mol^{-1}m^{3}s^{-1} resulting in slower rust precipitation

is considered. Compare this to Fig. 2c in the main manuscript for $k_r^{II\to III} = 0.1$. Reaction constant $k_r^{II\to o}$ was calculated according to our previous study [13] also for $i_a = 1$ µA/cm^2. Parameter t_c, i.e. the maximum distance to which rust can possibly precipitate in concrete from the original surface of a steel rebar, theoretically depends on the diffusivity of iron ions in concrete (which is affected by the cracking and porosity of concrete) but when considered in a reasonable range, it appears to have very limited impact on predicted t_{crit}. Thus, following the numerical results from our previous study [14], we set $t_c = 2$ mm.

**Parameter**	**Value**	**Unit**	**Source**
Young's modulus of rust E_r	500	MPa	Ref. [15]
Poisson's ratio of rust ν_r	0.4	-	Ref. [15]
Molar volume ratio of rust and steel κ	3.17	-	Ref. [13]
Diffusivity of iron ions in rust D_r	$7 \cdot 10^{-10}$	m^2 s^{-1}	Ref. [12]
Diffusivity of iron ions in water D_w	$7 \cdot 10^{-10}$	m^2 s^{-1}	Refs. [19, 20]
Reaction constant $k_r^{II\to o}$	$3.3 \cdot 10^{-3}$	s^{-1}	Ref. [13]
Reaction constant $k_r^{II\to III}$	0.1	mol^{-1}m^{3}s^{-1}	Ref. [19]
Maximum possible distance of rust in concrete pore space from rebar t_c	2	mm	Ref. [13]

Supplementary Table 1: Model parameters for iron transport in concrete, rust precipitation and its mechanical properties.

**Properties of autoclaved aerated concrete (AAC)**			
Tensile strength	Young's modulus	Concrete density	Total porosity
f_t	E_c	ρ_c	ϕ
MPa	MPa	kg/m^3	
0.52	675	400	0.85
0.68	1540	500	0.81
0.84	2200	600	0.77
1.00	3000	700	0.73

Supplementary Table 2: Model parameters of in considered RAAC specimen

Regarding the considered properties of autoclaved aerated concrete (AAC), it can be categorized by its density, which is directly linked to its porosity determined by

the amount of added aerating agent, with porosity in turn affecting the mechanical properties. As the inherent porosity of AAC is negligible compared to the porosity introduced by the presence of aerating agent [21], the total porosity can be directly linked to the concrete density as $p = (\rho_c - \rho_n)/\rho_n$, where ρ_c is the density of aerated concrete and ρ_n is the basic density of concrete with no added aerating agent. Thus, to study the critical corrosion penetration of AAC, we considered specimens with a density ranging between 400 - 700 kg/m^3 listed in Supplementary Table 2, while the typical density of construction AAC lies between 600 - 800 kg/m^3 [22]. Cabrillac et al. [21] measured ρ_n to be between roughly 2400 - 2800 kg/m^3. Thus, for the calculation of porosity, we considered $\rho_n = 2600$ kg/m^3. The values of Young's modulus E_c were chosen as the mean of the experimentally measured range reported by Narayanan and Ramamurthy [23] for density values ρ_n listed in Supplementary Table 2. For the calculations in Figs. 2c and 2d in the main manuscript, values of E_c were calculated as $E_c = 8\rho_c[\mathrm{kg/m^3}] - 2600$ which fits well RAAC Young's modulus values listed in Supplementary Table 2. Tensile strength was calculated according to the study of Michelini et al. [24] as $f_t[\mathrm{MPa}] = 0.0016\rho_c[\mathrm{kg/m^3}] - 0.1206$.

4 Calibration of the model

There are currently no available data on the critical corrosion penetration in aerated concrete. Thus, the proposed model, specifically exponent m in the expression $D_c = D_w\phi^m$ relating the diffusivity of iron ions in concrete D_c with concrete porosity ϕ had to be calibrated with data on t_{crit} in standard concrete. For this purpose, the exponent m was varied between 3 and 8 (see Supplementary Figure 2a) and a relative error $(t_{crit,calc} - t_{crit,exp})/t_{crit,calc}$ was evaluated for all considered tests. Exponent $m = 4.3$ was then obtained by minimizing the mean and median of the obtained errors. This choice generally leads to a good agreement of predicted and experimentally measured critical corrosion penetrations, as demonstrated in Supplementary Figure 2b. However, we can see that a couple of data lie far above the ideal $t_{crit,calc} = t_{crit,exp}$ line. This raises the question of whether these data may contain large experimental errors. To gain insight, we employ the observation of Vu and Stewart [16] that the water-to-cement ratio W/C and the thickness of concrete cover c have such a strong influence on the time-to-cracking T_{crit} of a standard concrete that it can be well-fitted by a power law $T_{crit} = A(c/(W/C))^B$, where A and B are constants. Cement paste porosity ϕ can be expected to be a monotonically increasing function of W/C and thus we propose an analogical dimensionless relation for critical corrosion penetration $t_{crit}/d = A(\phi c/d)^B$, where d is the diameter of the steel rebar. By fitting the model predictions, the optimal constant values minimizing the coefficient of determination were found to be $A = 0.18$ and $B = 0.59$. In Supplementary Figure 2c we can see that nearly all numerically predicted data and the majority of experimental data fall into the 25%-band around the proposed curve. This suggests that the worst experimental data outliers may be bearing a considerable experimental error. Also, the results indicate that the recovered curve $t_{crit} = 0.18(\phi c/d)^{0.59}$ can serve as an effective rough estimator for the critical penetration in standard concrete. However, let us stress here that with the suggested values of constants A and B, the proposed relation cannot be under any

circumstances expected to provide reasonable t_{crit} predictions for aerated concrete, as can be immediately observed from the comparison with the results presented in the main manuscript. Let us note here that the recovered value of m is affected by the choice of the parameters related to the iron transport in concrete and rust precipitation listed in Supplementary Table 1. However, the values of these parameters are not known for RAAC yet. The reaction constant $k_r^{II \rightarrow III}$ governing the rate of oxidation of Fe^{2+} to Fe^{3+} ions is especially influential. If lower value of $k_r^{II \rightarrow III} = 0.01$ mol^{-1}m^{3}s^{-1} meaning slower precipitation was considered, the value of exponent m would change to 5.5.

As there are currently no available data on the critical corrosion penetration in aerated concrete, data employed for the model calibration were collected from impressed current tests (i.e. a concrete specimen with uniformly corroding rebars under constant corrosion current density) conducted on samples from standard concrete of various porosity and mechanical properties from studies [16, 25–27] (all data are listed in Supplementary Table 3). Aldellaa [25] and Lu et al. [27] measured the compressive strength on cubic specimens, which was then converted to the cylindrical compressive strength $f_{c,cyl}$ by multiplication by 0.8. In both of these studies, the tensile strength of concrete f_t was not measured, so it was calculated in accordance with ACI-318-19 [28] recommendation as $f_t[\text{MPa}] = 0.56\sqrt{f_{c,cyl}[\text{MPa}]}$. For all considered tests, Young's modulus of concrete E_c was estimated according to ACI-318-19 [28] as $E_c[\text{MPa}] = 4700\sqrt{f_{c,cyl}[\text{MPa}]}$. The total porosity of cement paste was estimated from the reported water-to-cement ratio using the model of Powers and Brownyard [29]. It is also important to note that in the impressed current tests used to calibrate the proposed model (as listed in Supplementary Table 3), corrosion was initiated by the high concentration of chlorides to ensure uniform corrosion of the rebars. This is a standard approach used in impressed current tests. It is known that the presence of chlorides significantly increases the solubility of corrosion products [18], resulting in slower rust accumulation. In addition, unlike during carbonation-induced corrosion, the porosity of the concrete is not significantly affected. These, together with the choice of reaction constants, implicitly influence the calibration of the exponent m in the formula $D_c = D_w \phi^m$ describing the dependence of iron ion diffusivity on concrete porosity. As discussed previously, while carbonation-induced corrosion can be reasonably expected for RAAC panels, the presence of chlorides cannot be completely ruled out depending on the exposure conditions of the particular panel. If corrosion is carbonation-induced, then calibration to tests based on chloride-induced corrosion is likely to overestimate the recovered iron ion diffusivity in concrete. This means that the predicted critical corrosion penetration would also be overestimated and thus on the safe side.

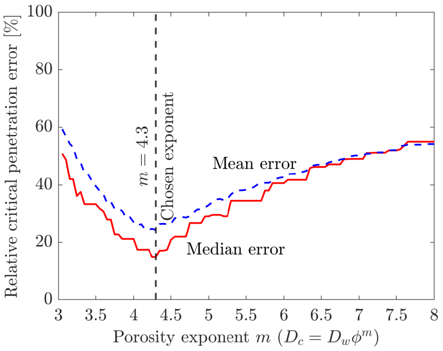

(a)

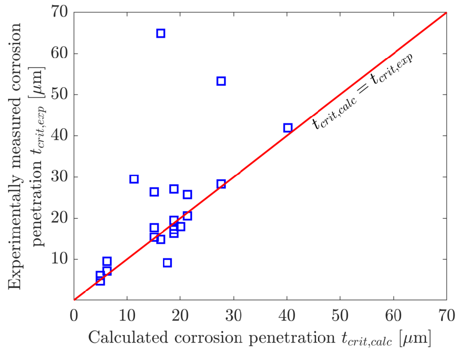

(b)

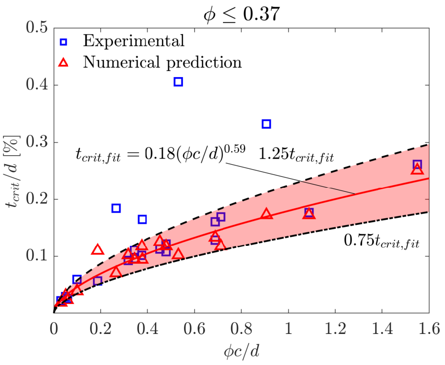

(c)

Supplementary Figure 2: **Calibration of the proposed model.** Iron ions diffusivity $D_c = D_w\phi^m$ in concrete is the function of concrete porosity, where D_w is the diffusivity of iron ions in water. It is a critical input of the model as it is responsible for the dependency of a critical corrosion penetration on porosity. Exponent m was calibrated from experimental data (a) by minimizing mean and median of the relative error $(t_{crit,calc} - t_{crit,exp})/t_{crit,calc}$ which provided $m = 4.3$. This choice leads to a good agreement of experimental and predicted critical corrosion penetration t_{crit} (b). t_{crit} is strongly influenced by a concrete porosity ϕ and concrete cover to rebar diameter ratio c/d. When t_{crit}/d, where d is a steel diameter, is plotted as the function of a non-dimensional parameter $\phi c/d$, dependency emerges and predicted data can be well fitted with $t_{crit}/d = 0.18(\phi c/d)^{0.59}$ (c). For standard concrete with $\phi \leq 0.37$, the majority of experimental data lies in the 25% range of this curve which can serve for rough quick estimates for standard concrete. Let us stress here that this estimate does not hold for aerated concrete.

Reber diameter	Concrete cover	Corrosion current density	Concrete tensile strength	Concrete compressive strength	Young's modulus	Water-to-cement ratio	Total porosity	Critical corrosion penetration
d	c	i_a	f_t	f_c	E_c	W/C	ϕ	$t_{crit,exp}$
mm	mm	$\mu A/cm^2$	MPa	MPa	MPa			µm
Data of Aldellaa [25]								
16	67	100	3.6	42.4	30600	0.38	0.09	26.4
16	67	100	3.0	29.6	25600	0.45	0.17	27.1
16	67	100	2.7	23.2	22600	0.55	0.26	28.3
16	67	100	2.1	14.4	17800	0.70	0.37	41.9
Data of Vu et al. [16]								
16	25	112.7	4.6	52.7	34100	0.45	0.17	29.5
16	50	123.6	4.6	52.7	34100	0.45	0.17	64.9
16	25	140.3	3.1	20.0	21000	0.50	0.22	17.7
16	50	128.1	3.1	20.0	21000	0.50	0.22	25.8
16	25	93.2	4.2	43.0	30800	0.50	0.22	15.3
16	50	106.3	4.2	43.0	30800	0.50	0.22	20.6
16	25	140.3	3.9	42.3	30600	0.58	0.29	18.0
16	50	86.8	3.9	42.3	30600	0.58	0.29	53.3
Data of Al-Harthy et al. [26]								
16	20	184	2.4	23.4	22700	0.37	0.08	9.5
16	10	172	2.4	23.4	22700	0.37	0.08	4.8
27	20	139	2.4	23.4	22700	0.37	0.08	7.1
27	10	116	2.4	23.4	22700	0.37	0.08	6.1
16	20	140	1.3	8.0	13300	0.59	0.3	16.3
16	10	153	1.3	8.0	13300	0.59	0.3	9.1
Data of Lu et al. [27]								
16	29.5	100	2.2	15.5	18500	0.54	0.26	19.5
16	29.5	150	2.2	15.5	18500	0.54	0.26	17.3
16	19.5	100	2.2	15.5	18500	0.54	0.26	14.8

Supplementary Table 3: Model parameters for simulated experimental impressed current tests from studies [16, 25–27] and experimentally recovered critical corrosion penetrations $t_{crit,exp}$.

Supplementary References

[1] Brent, R.P.: Algorithms for Minimization Without Derivatives. Courier Corporation, New York (2013)

[2] Kierzenka, J., Shampine, L.F.: A bvp solver based on residual control and the maltab pse. ACM Transactions on Mathematical Software (TOMS) **27**(3), 299–316 (2001)

[3] Liddell, M., Palmer, M., Rolf, A., Atkins, C., Brown, R., Goodier, C.: Reinforced Autoclaved Aerated Concrete (RAAC) Investigation and Assessment – Further Guidance. Technical Report April, The Institution of Structural Engineers (2023)

[4] Korec, E., Mingazzi, L., Freddi, F., Martínez-Pañeda, E.: Predicting the impact of water transport on carbonation-induced corrosion in variably saturated reinforced concrete. Materials and Structures **57**(4), 1–16 (2024)

[5] Korec, E., Jirásek, M., Wong, H.S., Martínez-Pañeda, E.: Phase-field chemo-mechanical modelling of corrosion-induced cracking in reinforced concrete subjected to non-uniform chloride-induced corrosion. Theoretical and Applied Fracture Mechanics **129**(December 2023), 104233 (2024)

[6] Stefanoni, M., Angst, U.M., Elsener, B.: Kinetics of electrochemical dissolution of metals in porous media. Nature Materials **18**(9), 942–947 (2019)

[7] Andrade, C.: Role of Oxygen and Humidity in the Reinforcement Corrosion. In: Proceedings of the 75th RILEM Annual Week 2021: Advances in Sustainable Construction Materials and Structures, pp. 316–325. Springer, Cham, Switzerland (2023)

[8] Otieno, M., Beushausen, H., Alexander, M.: Prediction of corrosion rate in reinforced concrete structures - A critical review and preliminary results. Materials and Corrosion **63**(9), 777–790 (2012)

[9] Otieno, M., Beushausen, H., Alexander, M.: Chloride-induced corrosion of steel in cracked concrete - Part I: Experimental studies under accelerated and natural marine environments. Cement and Concrete Research **79**, 373–385 (2016)

[10] Andrade, C.: Steel corrosion rates in concrete in contact to sea water. Cement and Concrete Research **165**(January), 107085 (2023)

[11] Walsh, M.T., Sagüés, A.A.: Steel corrosion in submerged concrete structures-part 1: Field observations and corrosion distribution modeling. Corrosion **72**(4), 518–533 (2016)

[12] Ansari, T.Q., Luo, J.L., Shi, S.Q.: Modeling the effect of insoluble corrosion products on pitting corrosion kinetics of metals. npj Materials Degradation **3**(1), 1–12

(2019)

[13] Korec, E., Jirásek, M., Wong, H.S., Martínez-Pañeda, E.: Unravelling the interplay between steel rebar corrosion rate and corrosion-induced cracking of reinforced concrete. Submitted to Cement and Concrete Research (2023)

[14] Korec, E., Jirásek, M., Wong, H.S., Martínez-Pañeda, E.: A phase-field chemo-mechanical model for corrosion-induced cracking in reinforced concrete. Construction and Building Materials **393**, 131964 (2023)

[15] Zhao, Y., Jin, W.: Chapter 2 - Steel Corrosion in Concrete. In: Zhao, Y., Jin, W. (eds.) Steel Corrosion-Induced Concrete Cracking, pp. 19–29. Butterworth-Heinemann, Oxford (2016)

[16] Vu, K., Stewart, M.G., Mullard, J.: Corrosion-induced cracking: Experimental data and predictive models. ACI Structural Journal **102**(5), 719–726 (2005)

[17] Zhang, W., Chen, J., Luo, X.: Effects of impressed current density on corrosion induced cracking of concrete cover. Construction and Building Materials **204**, 213–223 (2019)

[18] Furcas, F.E., Lothenbach, B., Isgor, O.B., Mundra, S., Zhang, Z., Angst, U.M.: Solubility and speciation of iron in cementitious systems. Cement and Concrete Research **151**(September 2021), 106620 (2022)

[19] Stefanoni, M., Zhang, Z., Angst, U.M., Elsener, B.: The kinetic competition between transport and oxidation of ferrous ions governs precipitation of corrosion products in carbonated concrete. RILEM Technical Letters **3**, 8–16 (2018)

[20] Leupin, O.X., Smart, N.R., Zhang, Z., Stefanoni, M., Angst, U.M., Papafotiou, A., Diomidis, N.: Anaerobic corrosion of carbon steel in bentonite: An evolving interface. Corrosion Science **187**(December 2020), 109523 (2021)

[21] Cabrillac, R., Fiorio, B., Beaucour, A.L., Dumontet, H., Ortola, S.: Experimental study of the mechanical anisotropy of aerated concretes and of the adjustment parameters of the introduced porosity. Construction and Building Materials **20**(5), 286–295 (2006)

[22] Goodier, C., Cavalaro, S., Lee, K., Casselden, R.: Durability variations in reinforced autoclaved aerated concrete (RAAC) – extended abstract. MATEC Web of Conferences **361**, 06005 (2022)

[23] Narayanan, N., Ramamurthy, K.: Structure and properties of aerated concrete: A review. Cement and Concrete Composites **22**(5), 321–329 (2000)

[24] Michelini, E., Ferretti, D., Pizzati, M.: The influence of density on the fracture energy of AAC: From experimental investigation to the calibration of a cohesive

law. Construction and Building Materials **400**(April), 132547 (2023)

[25] Aldellaa, I.: Corrosion-induced cracking in reinforced concrete. Phd thesis, University of Glasgow (2024)

[26] Al-Harthy, A.S., Stewart, M.G., Mullard, J.: Concrete cover cracking caused by steel reinforcement corrosion. Magazine of Concrete Research **63**(9), 655–667 (2011)

[27] Lu, C.H., Jin, W.L., Mao, J.H.: Experimental investigation of corrosion-induced cover cracking in reinforced concrete structures. Advanced Materials Research **197-198**(4), 1690–1693 (2011)

[28] CODE-318-19 (22); Building Code Requirements for Structural Concrete and Commentary. Technical report, American Concrete Institute, Indianapolis, USA (2022)

[29] Powers, T.C., Brownyard, T.L.: Studies of the physical properties of hardened portland cement paste. American Concrete Institute, ACI Special Publication **SP-249**(October 1946), 265–617 (1946)
